# Network-Based Integrative Analysis of Genomics, Epigenomics and Transcriptomics in Autism Spectrum Disorders

**DOI:** 10.3390/ijms20133363

**Published:** 2019-07-09

**Authors:** Noemi Di Nanni, Matteo Bersanelli, Francesca Anna Cupaioli, Luciano Milanesi, Alessandra Mezzelani, Ettore Mosca

**Affiliations:** 1 Institute of Biomedical Technologies, Italian National Research Council, Via Fratelli Cervi 93, 20090 Segrate (MI), Italy; 2 Department of Industrial and Information Engineering, University of Pavia, Via Ferrata 5, 27100 Pavia, Italy; 3 Department of Physics and Astronomy, University of Bologna, Via B. Pichat 6/2, 40127 Bologna, Italy; 4 National Institute of Nuclear Physics (INFN), 40127 Bologna, Italy

**Keywords:** autism spectrum disorders, biological networks, genomics, multi-omics, network diffusion, data integration

## Abstract

Current studies suggest that autism spectrum disorders (ASDs) may be caused by many genetic factors. In fact, collectively considering multiple studies aimed at characterizing the basic pathophysiology of ASDs, a large number of genes has been proposed. Addressing the problem of molecular data interpretation using gene networks helps to explain genetic heterogeneity in terms of shared pathways. Besides, the integrative analysis of multiple omics has emerged as an approach to provide a more comprehensive view of a disease. In this work, we carry out a network-based meta-analysis of the genes reported as associated with ASDs by studies that involved genomics, epigenomics, and transcriptomics. Collectively, our analysis provides a prioritization of the large number of genes proposed to be associated with ASDs, based on genes’ relevance within the intracellular circuits, the strength of the supporting evidence of association with ASDs, and the number of different molecular alterations affecting genes. We discuss the presence of the prioritized genes in the SFARI (Simons Foundation Autism Research Initiative) database and in gene networks associated with ASDs by other investigations. Lastly, we provide the full results of our analyses to encourage further studies on common targets amenable to therapy.

## 1. Introduction

Autism spectrum disorders (ASDs) are among the most common neurodevelopmental disorders. ASDs are characterized by impaired social interactions, repetitive behavior, and restricted interests, and they are often comorbidities with other conditions such as epilepsy, mental retardation, inflammation, and gastrointestinal disorders. Despite the fact that the high heritability of ASDs is well established, the exact underlying causes are unknown in at least 70% of the cases [1]. Large genome-wide association studies (GWAS), Copy Number Variation (CNV) testing and genome sequencing yielded many non-overlapping genes, a fact that underlines the complex genetic heterogeneity of ASDs [1] and reflects the architecture of intracellular networks, in which several possible combinations of genetic variations are likely to lead to a common pathological phenotype [2,3].

The identification of the key molecular pathways that link many ASDs-causing genes is of prominent importance in developing therapeutic interventions [1]. In this context, network-based and pathway-based analyses provide functional explanations to non-overlapping genes and narrow the targets for therapeutic intervention [4]. The rich functional pathway information emerging from such analyses might unearth common targets that are amenable to therapy [1].

One of the challenges that network-based analyses face is the identification of the so-called “disease modules,” i.e., gene networks associated with diseases [2]. Under the hypothesis of the “omnigenic model,” gene regulatory networks are so interconnected that a large number of genes is liable to affect the function of core genes, i.e., those whose variations are strongly related to disease [3].

The analysis of the human interactome—the complex web of molecular interactions occurring within human cells—is challenging due to its size (e.g., 10^4^ genes and 10^5^ interactions), and several approaches have been proposed [5]. In the last few decades, the mathematical machinery of network diffusion (ND)—also referred to as network propagation—has been exploited to address several problems in biological data analysis, thanks to its ability to quantify network proximity between query network nodes (e.g., genes) and to simultaneously consider all the possible network paths among them [6]. When applied to studying the large number of genes proposed to be associated with a pathology like ASDs (shortly, disease genes), ND amplifies the relevance of those disease genes that are in close network proximity with other disease genes. In addition, ND predicts the importance of other genes not known a priori but that will probably act as “linkers” (or “silent players”), because they occupy a relevant network position in relation to the network location of disease genes.

In a previous study by our group, the application of ND to genes associated with ASDs from genetic data led to the identification of gene networks and pathways particularly enriched in disease genes [7]. Interestingly, several genes predicted as relevant in such study are now included in the SFARI (Simons Foundation Autism Research Initiative) Gene database [8], which provides curated information on all known human genes associated with ASDs.

In addition to genetics, several reports have suggested a role for epigenetic mechanisms in ASD etiology [9,10]. Recent studies have also demonstrated the utility of integrating gene expression with mutation data for the prioritization of genes disrupted by potentially pathogenic mutations [11,12]. More generally, the integrative analysis of multiple omics has emerged as an approach to provide a more comprehensive view of a disease [13,14].

While the analysis of epigenomics and transcriptomics from brain-derived samples can provide important insights into the potential mechanisms of disease etiology, there are relevant limitations with these types of studies (e.g., the quality of autopsy-derived tissue, sample size, influence of life experience, and cause of death) [10]. These barriers have been overcome by analyzing blood samples, and recent blood-based works have shown the usefulness of this alternative approach to gather insights into ASDs [10,15,16,17].

In this manuscript, we describe a network-based integrative meta-analysis of the results which have emerged from several studies on ASDs, based on genomics, epigenomics, and transcriptomics. Firstly, following the hypothesis of the omnigenic model [3], we analyzed genetic data to introduce a graduated scale of gene relevance in relation to core genes for ASDs. Subsequently, we identified a gene network significantly enriched in genes supported by one or more of the considered evidence (genomics, epigenomics, and transcriptomics). The gene network involves genes that participate in several pathways relevant to ASDs, which we have distinguished by type (or types) of alteration from which they are affected. Collectively, our network-based meta-analysis provides a prioritization of the large number of genes proposed to be associated with ASDs, based on genes’ relevance within the intracellular circuits, the strength of the supporting evidences of association with ASDs, and the number of different molecular alterations affecting genes. We discuss the presence of the prioritized genes in the SFARI database and in gene networks associated with ASDs by other studies [18,19,20,21,22]. Lastly, we provide the full results of our analyses to encourage further studies on common targets amenable to therapy.

## 2. Results

Firstly, we describe the results obtained regarding the genes associated with ASDs on the basis of genomics. We collected these genes from the SFARI Gene database [8], two recent large studies [23,24], and a series of previous studies summarized by Mosca et al. [7], for a total of 1133 genes (Table 1). Following the criteria adopted by SFARI, we distinguished between the genes with the strongest genomics evidence (334 genes, the “genomics-major” group) from the others (799, the “genomics-minor”).

Subsequently, we could describe the multi-omics analysis in which we also considered evidences emerged in studies that focused on epigenomics [10] and transcriptomics [15,25,26,27]. Additionally in these cases, we distinguished between the genes with the strongest evidences (“epigenomics-major” and “transcriptomics-major”) from the others (“epigenomics-minor” and “transcriptomics-minor”), following the indications of the corresponding studies from which we collected the data (Table 1).

### 2.1. Genomics Analysis

Recently, the “omnigenic model” was proposed to explain the inheritance of complex diseases [3]. In this model, the genes whose genetic damage tend to have the strongest effects on disease risk are considered core genes, while those genes that have a minor impact on disease risk are designated as peripheral. The number of peripheral genes may be large as a consequence of the multiple ways in which these genes may interact with core genes throughout cell regulatory networks. Importantly, such classification may be on a graduated scale rather than simply binary [3].

In this context, ND provides an opportunity to define quantitatively the degree of peripherality of all genes in relation to “seed” genes, exploring all possible network paths among genes in intracellular networks. We applied ND on the human interactome (high confidence functional and biophysical interactions catalogued in STRING), considering as seeds: the core genes for ASDs, among which we included those classified in SFARI as “syndromic,” “high confidence,” “strong candidate,” “suggestive evidence,” and “syndromic minimal evidence,” for a total of 334 genes; other 799 genes proposed to have a role in ASDs (Supplementary Table S1).

**Figure 1:**
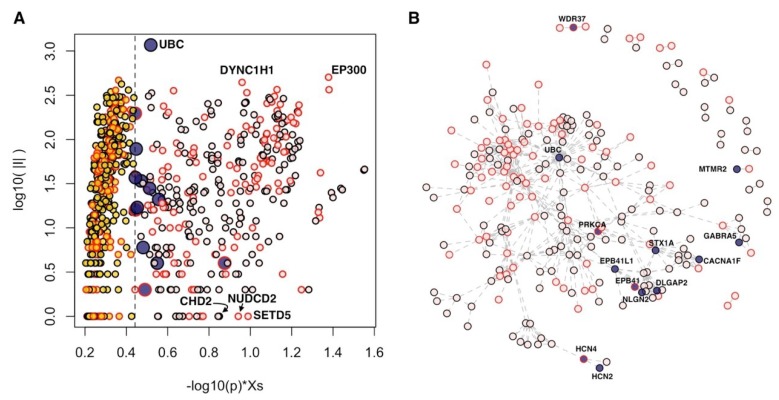
Genes in network proximity to the core genes of autism spectrum disorders (ASDs). (**A**) Diffusion score (Xs) normalized by its empirical p-value (horizontal axis) and number of interactions (|I|, vertical axis); only genes with *p* < 0.05 are shown. (**B**) Connected components of “core+13” network. (**A**,**B**) Blue points: 13 genes of “core+13”; pink points: core genes; yellow points: Significant genes outside “core+13” genes; red border of points: Genes supported at transcriptomic and/or epigenetic levels.

We found several genes with a significant network proximity to core genes (Figure 1A, Supplementary Table S1). From a topological point of view, among these genes, we found both hubs (genes that establish many interactions, such as UBC, ubiquitin C; DYNC1H1, dynein cytoplasmic 1 heavy chain 1; and EP300, E1A binding protein p300) and genes with a lower number of connections (e.g., CHD2, chromodomain helicase DNA binding protein 2; NUDCD2, NudC domain containing 2; and SETD5, SET domain containing 5), which are nevertheless important for the information flow within the network (Figure 1A).

Interestingly, 13 genes obtained scores comparable to those of core genes (Figure 1). These results indicate that these 13 genes closely interact with the core genes, and, in almost all cases, the number of interactions that these genes establish with the core genes is significant (Table 2, Supplementary Figure S1). From now on, we will call the set of core genes and the 13 genes closely related to the core genes as “core+13.” In the core+13 gene network, the 13 genes act as linkers between groups of core genes not directly connected with each other; for instance, *WDR37* (WD repeat domain 37) links *PACS1* (phosphofurin acidic cluster sorting protein 1) and *PACS2* (phosphofurin acidic cluster sorting protein 2). The resulting largest connected component involves 204 genes, while the remaining 143 genes are mostly isolated or form very small modules of two or three genes.

We checked whether any of these 13 genes, currently not included in the highest categories of SFARI, are nevertheless classified in other categories corresponding to a lower degree of evidence or have been reported in other network-based analyses of ASDs data. We found that six genes belong to the categories designated as “minimal evidence” or “hypothesized but untested,” and eight genes were proposed as part of gene networks associated with ASDs (Table 2).

The association with ASDs for 12 of the 13 genes is supported at genomic level. In addition, *HCN4* (hyperpolarization activated cyclic nucleotide gated potassium channel 4) was found with epigenetic modifications in a study of Andrews et al. [10], while *PRKCA* (protein kinase C alpha) was found both epigenetically modified [10] and differentially expressed [26]. *WDR37* does not have supporting evidences at genomic level, but it was found differentially expressed [25].

### 2.2. Multi-Omics Analysis

**Figure 2:**
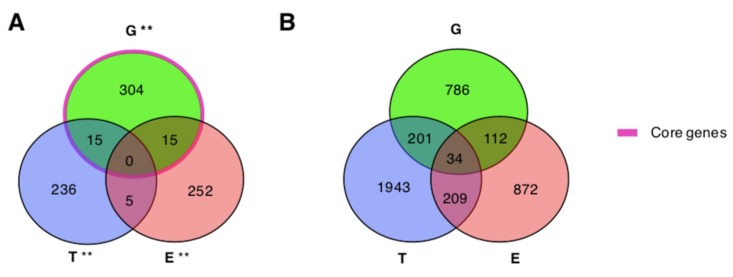
Overlaps among genes associated with ASDs by genomics, epigenomics, and transcriptomics. (**A**,**B**) G: Genomics; E: Epigenomics; T: Transcriptomics. ** major.

We assessed the significance of the overlaps among the lists of genes associated with ASDs by genomics, epigenomics, and transcriptomics evidences. We observed significant overlaps between the list of genes from genomics and those supported by epigenomics or transcriptomics (Table 3). The intersection among the three gene lists consists of 40 genes, 34 of which are included in the considered interactome (shortly “shared”) (Figure 2). Out of the shared genes, 26 do not interact directly with any other shared gene, while eight genes form three connected components composed of: *DYNC1H1*, *TRAPPC6B* (trafficking protein particle complex 6B), *TRAPPC9* (trafficking protein particle complex 9) and *CSNK1D* (casein kinase 1 delta); *GNAS* (GNAS complex locus) and *PRKCA* (protein kinase C alpha); *EP400* and *TRRAP* (transformation/transcription domain associated protein) (Supplementary Figure S2).

In order to find modules of functionally related genes supported by one or more types of evidences (“layers” from now on), we used ND (see methods) and obtained a final diffusion score that summarized the relevance of each gene in relation to its location in the interactome and its network proximity to other genes associated with ASDs in one or more layers (genomics, epigenomics, and transcriptomics). The higher the final diffusion score, the closer the gene to ASDs genes in one or more of the considered layers.

**Figure 3:**
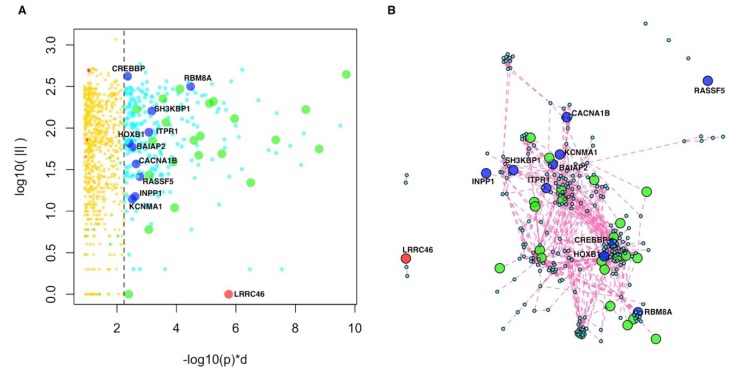
Integrative multi-omics analysis. (**A**) Global network diffusion scores (horizontal axis) and number of interactions (vertical axis) of the top ranking genes; the vertical dashed line separates the top 275 genes belonging to the INT-MODULE (higher scores, on the right) from the other genes (lower scores, on the left). (**B**) Network of the top 275 genes (INT-MODULE). Green circles: shared genes; blue circles: Genes included in SFARI categories 4 and 5; red circle: *LRRC46*.

At the top of the resulting genome-wide ranking, we found genes with significant scores (Figure 3, Supplementary Table S2). To assess whether these highly ranked genes formed significantly connected gene modules, we used network resampling [28] and found a multi-omics integrative gene module (INT-MODULE) involving a total of 275 genes (Supplementary Figure S3). The largest connected component (266 genes) of the INT-MODULE connected 22 shared genes which do not establish direct interactions with each other if considered in isolation (Figure 3).

We compared the INT-MODULE with gene networks proposed by other studies on ASDs and found that 157 genes occurred in at least one of such networks (Supplementary Table S3). In addition to the 144 INT-MODULE genes occurring among the highest SFARI categories, we found that 10 genes are classified as “minimal evidence” and “hypothesized but untested” in SFARI (Table 4, Supplementary Figure S4), and seven of these 10 genes were reported by other network-based analyses (Table 4). The INT-MODULE includes also *LRRC46* (leucine rich repeat containing 46), the only gene of the module that does not occur in any of the input gene lists (Figure 3, Supplementary Figure S4).

**Figure 4:**
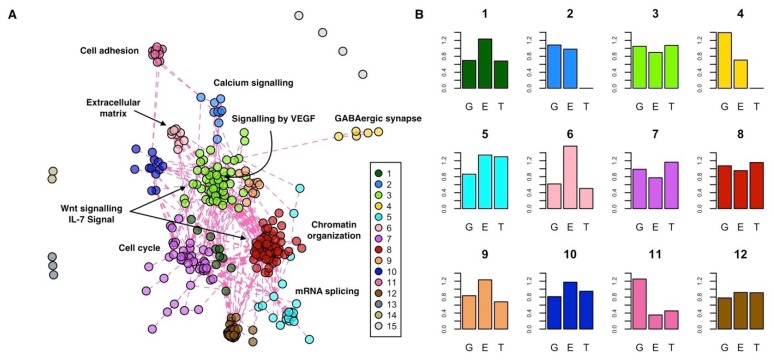
Functional characterization of the INT-MODULE. (**A**) Topological clusters; #1–12: Clusters of the largest connected component; #13,14: Two clusters of three and two genes, respectively; #15: The remaining four genes. (**B**) Enrichment (vertical axis) of each cluster in terms of genes supported by genomics (G), epigenomics (E), and transcriptomics (T): A value of 1 indicates the same proportion within the cluster and in the whole INT-MODULE.

To functionally characterize the INT-MODULE, we partitioned its largest connected component (266 genes) in topological clusters and assessed both the enrichment of each cluster in terms of molecular pathways and the types of evidences associated with each cluster (Supplementary Tables S2, S4–S6). We explored several community detection strategies and found the highest modularity with a partition of 12 clusters (Figure 4A, Supplementary Figure S5).

The two largest clusters are composed of 61 (cluster #8) and 53 (cluster #3) genes, and they are characterized by a similar proportion of supporting evidences (Figure 4B). These two central clusters contain genes that are part of the same pathways, such as the Wnt signaling pathway (#8: *q* = 1.83 × 10^−2^; #3: *q* = 1.79 × 10^−5^) and IL-7 signal transduction (#8: *q* = 4.54 × 10^−2^; #3: *q* = 6.02 × 10^−4^), but they are also marked by specific pathways. In particular, among the pathways specifically enriched in cluster #8 and #3, we found chromatin organization (*q* = 1.27 × 10^−27^) and signaling by VEGF (*q* = 5.41 × 10^−23^), respectively. Cluster #7 (41 genes) is the most enriched in differentially expressed genes and significantly associated with pathways involved in cell cycle processes. Cluster #5 is mainly enriched in genes associated with epigenetic and transcriptional changes, and it is marked by mRNA splicing (*q* = 1.92 × 10^−12^). Cluster #6 is particularly enriched in genes with epigenetic changes and associated it with extracellular matrix organization (*q* = 6.10 × 10^−5^). Cluster #2 (nine genes) is supported at the genomics and epigenomics levels and is enriched in genes of the calcium signaling pathway (*q* = 4.88 × 10^−12^). Lastly, clusters #11 and #4 are composed of genes associated with ASDs mainly at the genetic level, which, respectively, control the GABAergic synapse (#4: *q* = 3.90 × 10^−6^) and encode for cell adhesion molecules (#11: *q* = 1.58 × 10^−5^) active in the neuronal system.

## 3. Discussion

The integrative analysis of gene related evidence (e.g., DNA polymorphism or mutations, epigenetic changes, transcriptional variations) and gene–gene interaction evidences allows for the extraction of otherwise hidden patterns. In this context, it is worth noting that 68 genes that we proposed as relevant to ASDs in a previous network-based analysis of genetic data are now classified in SFARI (Supplementary Table S7).

In light of the utmost importance of jointly analyzing omics data and intracellular networks, Boyle et al. [3] proposed an explanation for the large number of genes that may be involved in a complex disease as the result of the highly interacting nature of molecular networks. Following the omnigenic model [3], we considered as core genes of ASDs those whose variations are highly scored in SFARI and quantified, by means of network proximity, the degree of peripherality of all other genes in relation to the core genes. This analysis led to the identification of 13 genes significantly connected with the core genes. The strong functional relationship we found between these 13 genes and the core genes suggests that even the former can play an important role in ASDs.

As for the 13 predicted genes (Table 2) that closely interact with the core genes of ASDs, they mainly belong to different neuronal pathways and are especially involved in synaptic function and plasticity that, if impaired, could actively contribute to the pathogenesis of ASDs and/or to their comorbidities. Genes encoding for the ion channel were found among these genes, and the role of various ion channel gene defects (channelopathies) is known in the pathogenesis of ASDs. For instance, *HCN2* and *HCN4* belong to the hyperpolarization-activated cyclic nucleotide-gated (HCN) channels family, encoding for non-selective voltage-gated cation channels, and they are strongly expressed in the brain. These channels establish the slow native pacemaker currents contributing to membrane resting potentials, input resistance, dendritic integration, synaptic transmission, and neuronal excitability. Interestingly, it seems that *SHANK3*, strongly linked to ASDs, works in organization of HCN-channels [29] and that its expression negatively influences those of *HCN2* [30], so variations in the *SHANK3* gene are reflected in pacemaker current abnormalities. In addition, variants in *HCN1*, another member of the HCN family, were detected in patients with epileptic encephalopathy and clinical features of Dravet syndrome, intellectual disability, and autistic features [31].

Some of the predicted genes, such as *EPB41* and *EPB41L1*, take part in cytoskeleton and synaptic structures. *EPB41* is the founding member of the large family of proteins that associate with membrane proteins and cytoskeleton and in neurons is involved in protein–protein interactions at synaptic level. It interacts with NRXN1 and NRXN2, as well as NLGN1, -2, -3, and -4X. These proteins act at the presynaptic and post synaptic level and causative variations in *NRXN1*, -*2* [32,33], as well and *NLGN2* (also in core+13 gene set), -*3,* and -*4X* [34,35] have already been described in ASDs. Furthermore, EPB41L1 (highly expressed in the brain) and the ionotropic glutamate receptor GRIA1, were listed in the 13 predicted and in core genes, respectively, interact thus contributing to glutamate neurotransmission. An alteration of glutamate neurotransmission was found in ASDs. Interestingly, EPB41L1 is associated with mental retardation, deafness autosomal dominant 11 and autosomal dominant non-syndromic intellectual disability.

Then again, DLGAP2 is a member of the postsynaptic density proteins (as SHANK3), probably involved in molecular organization of synapses and signaling in neuronal cells, with implications in synaptogenesis and plasticity. In particular, DLGAP2 could be an adapter protein linking the ion channel to the sub-synaptic cytoskeleton. Animal models demonstrated that *DLGAP2* has key role in social behaviors and synaptic functions [36]. Case studies also report rare *DLGAP2* duplications in ASDs [37,38,39]. Then again, the *DLGAP2* gene has an important paralog, *DLGAP1*, already associated with ASDs. DLGAP1 proteins interact with other ASDs-associated proteins such as DLG1, DLG4, SHANK1, SHANK2 and SHANK3 [18]. Moreover, the analysis of rare copy number variants in ASDs found numerous de novo and inherited events in many novel ASDs genes including *DLGAP2* [22].

Among the 13 predicted genes, syntaxin-1A (*STX1A*) is also involved in synaptic signaling. This gene encodes for part of complex of proteins mediating fusion of synaptic vesicles with the presynaptic plasma membrane. A dysregulation of *STX1A* expression [40,41,42] has been reported in high functioning autism and Asperger syndrome. A significant association between three *STX1A* SNPs (Single Nucleotide Polymorhpisms) and Asperger syndrome was recently described. These SNPs could alter transcription factor binding sites both directly and through other variants in linkage disequilibrium [43].

The list of predicted genes includes *GABRA5*. It transcribes for the subunit 5 of GABA receptor alpha whose reduced expression and reduced protein level have been described in autism [44], and the SNPs of this gene are biomarkers of symptoms and developmental deficit in Han Chinese with autism [45]. The inclusion of this gene in the core list strengthens the evidences of imbalance between excitatory and inhibitory neurotransmission in ASDs and abnormalities in glutamate and GABA signaling as possible causative pathological mechanisms of ASDs.

Few of these predicted genes encode for proteins involved in non-neuronal specific signaling pathways, which are also important for ASDs: *PRKCA, WDR3*7 and *UBC*. PRKCA regulates many signaling pathways such as cell proliferation, apoptosis, differentiation, tumorigenesis, angiogenesis, platelet function, and inflammation. A meta-analysis performed on the de novo mutation data of 10,927 individuals with neurodevelopmental disorders found an excess of missense variants in the *PRKCA* gene [46]. The *WDR37* gene encodes a member of a protein family that is involved in many cellular processes such as cell cycle progression, signal transduction, apoptosis, and gene regulation. WDR37 is a nuclear protein ubiquitous expressed and particularly abundant in the cerebellum and whole brain. There are no direct evidences for ASDs development and WDR37—however, recently, it has been demonstrated that WDR47 shares functional characteristics with PAFAH1B1, which causes lissencephaly. PAFAH1B1 also constitutes a key protein-network interaction node with high-risk ASDs genes expressed in the synapse that can impact synaptogenesis and social behavior [47].

Our analysis confirms the importance of the X-linked gene in the aetiopathogenesis of ASDs. Mutations of *CACNA1F* (located at Xp11.23) mainly cause X-linked eye disorders. Since the role of various ion channel gene defects (channelopathies) in the pathogenesis of ASDs is becoming evident, the deep resequencing of these functional genomic regions has been performed. These studies revealed potentially causative rare variants contributing to ASDs in *CACNA1F*. Then again, *CACNA1D*, an important paralog of *CACNA1F*, displayed de novo missense variants in ASDs probands from the Simons Simplex Collection [48,49]. Moreover, the gene being X-linked could contribute to the sex bias of ASDs.

Out of the 13 genes tightly interconnected with the core, the occurrence in SFARI (six genes in “minimal evidence” or “hypothesized but untested”), the inclusion in networks associated with ASDs by other studies (eight genes) and the presence of epigenetics and/or transcriptional changes modified in ASDs patients vs controls (four genes), constitute further evidences in favor of these genes. A similar reasoning can be extended to peripheral genes, for which we proposed a graduated scale of relevance in relation to the core genes. To this aim, we provided the full results.

Overall, we observed a significant overlap between the lists of genes associated with ASDs by studies of genomics, as well as by studies of epigenomics and transcriptomics from blood samples, with a total of 40 genes supported by all the three types of evidences (of which 34 had high confidence functional interactions). We also observed that a significant number of the core+13 genes has been reported as epigenetically and/or transcriptionally modified in ASDs patients. The observation that different types of alterations refer to the same genes further stresses the role of these genes in ASDs. These results are in line with those of previous studies that suggested the potential role of genetic factors in contributing to DNA methylation differences in ASDs [10]. Moreover, blood-derived epigenetic changes observed in genes whose sequence variations are associated with ASDs are more likely to have a common function across tissues compared to those not related to genetic changes [17].

The existence of molecular relations between altered genes increases the likelihood that such alterations have a role in ASDs, suggesting molecular pathways that encompass such genes and functional relations among different types of alterations. Our analysis highlighted a network of 275 genes, which is strongly supported by genomics, epigenomics, and transcriptomics. Importantly, this network gathers 22 genes not directly linked to each other but supported by all three types of evidences. Interestingly, 157 of the INT-MODULE genes were proposed by other network-based studies on ASDs, different in terms of input data and analysis approach. A total of 144 genes belong to the highest scoring SFARI categories. Ten other genes of the network are currently classified in SFARI as “minimal evidence” and “hypothesized but untested” and are also supported by epigenomics and/or transcriptomics. Therefore, they deserve special attention among the genes of such categories.

The largest connected component of the network (275 genes) can be partitioned in 12 subgroups or topological sub-modules. This analysis suggests a different role of the sub-modules by function and by association with one or more types of alterations. For example, cluster #3, equally supported by all the three types of evidence, includes genes that belong to inflammatory mediator regulation of transient receptor potential (TRP) channels. Inflammation and immune system dysfunctions are in comorbidity with ASDs, and TRP canonical channel 6 (TRPC6) is emerging as a functional element for the control of calcium currents in immune-committed cells and target tissues, influencing leukocytes tasks. Interestingly, TRPC6 is also involved in neuronal development and variants in the *TRPC6* gene (within core gene) were found in patients with ASDs. Moreover, MeCP2, a transcriptional regulator whose mutations cause Rett syndrome, was found abundant in a TRPC6 promoter region resulting a transcriptional regulator of this gene [50] TRPC6, in turn, activates neuronal pathways, including BDNF, CAMKIV, Akt, and CREB signaling pathways, also involved in ASDs [51].

The prioritization of genes in terms of causality is a relevant challenge, especially in a complex and multi-genic disorder like ASDs. Nevertheless, it is possible to distinguish, among the functional themes highlighted by our integrative analysis, possible causative pathways considering their function and/or alteration type. For example, genes of clusters #4 and #11 mainly display genetic alterations and participate, respectively, in neuronal cell adhesion and GABAergic synapses, pathways already associated with brain morpho-functional abnormalities in ASDs.

Our integrative analysis of the large number of genes reported by studies on ASDs that focused on genomics, epigenomics, and transcriptomics prioritized a series of genes interconnected by functional relations and associated with one or more types of molecular alteration. Since this rich information might unearth common targets that are amenable to therapy [1], we have provided the full results of our network-based meta-analyses.

## 4. Materials and Methods

### 4.1. Molecular Interactions

Molecular interactions were collected from the STRING database [52] for a total of 12,739 genes and 355,171 links with high confidence (score ≥ 700). Native identifiers were mapped to Entrez Gene [53] identifiers. In case multiple proteins mapped to the same gene identifier, only the pair of gene identifiers with the highest STRING confidence score was considered.

### 4.2. Genomics

Genes associated with ASDs on the basis of genomics evidences were collected from the SFARI Gene database [8] and previous studies [7,23,24]. The SFARI Gene scoring system classifies genes on the basis of the strength of the supporting evidences as: “Syndromic” (S), “high confidence” (1), “strong candidate” (2), “suggestive evidence” (3), “minimal evidence” (4), “hypothesized but untested” (5), and “Evidence does not support a role” (6). Genes classified as S, 1, 2, 3, 1S, 2S, 3S, and 4S were assigned to the genomics-major evidence group.

Genes belonging to the genomics-minor group were collected from Mosca et al. [7], in which genes associated with SNPs, mutations, and CNV emerging from several large studies were reported, the meta-analysis study of GWAS of over 16,000 individuals with ASDs [23], and the whole-exome sequencing study of rare coding variation in 3871 autism cases and 9937 ancestry-matched or parental controls [24]. Native gene identifiers were converted to Entrez Gene identifiers [53].

### 4.3. Epigenomics

Genes associated with ASDs at the epigenomics level were collected from a previous study [10] in which the authors performed a case-control meta-analysis of blood DNA methylation among two large case-control studies of autism (796 ASDs cases and 868 controls) using METAL software [54] on the probes that were present in both studies. All genes found by their meta-analysis with *p* < 10^−3^ were assigned to epigenomics-major group, while the genes with 10^−3^≤ *p* < 5 × 10^−3^ were assigned to epigenomics-minor. Native gene identifiers were converted to Entrez Gene [53] identifiers.

### 4.4. Transcriptomics

Genes associated with ASDs at transcriptomics level were collected from the four studies [15,25,26,27] reported in [55], in which the original authors generated blood-based gene expression profiles from microarray experiments with sample sizes greater than 40 and provided list of differentially expressed genes. Following the approach by [55], genes reported as differentially expressed in at least two studies were assigned to the transcriptomics-major group, while the other differentially expressed genes were assigned to the transcriptomics-minor group. Native gene identifiers were converted to Entrez Gene [53] identifiers, and only genes occurring in STRING network were considered in network-based analyses.

### 4.5. Gene Prioritization Based on Network Diffusion

Network diffusion (ND) was performed using an approach previously described [7,28,56,57]. A genes-by-layers input matrix $X_{0}=\left(x_{1},\;x_{2},\;x_{3}\right)$ was defined where each element $x_{ij}$ was set to: 1 if the gene *i* was member of a “-major” group in layer *j*; 0.5 if the gene *i* was member of a “-minor” group in layer *j*; and 0 if the gene was *i* was not associated with ASDs in layer *j*. ND was applied to $X_{0}$ using the genome-wide interactome represented by the symmetric normalized adjacency matrix **W**, according to the following iterative procedure:

$$X_{t+1}=\alpha WX_{t}+\left(1-\alpha \right)X_{0}$$

$$X^{ss}=\underset{t\to \infty }{\lim}X_{t}$$

where α∈(0, 1) is a scalar that weights the relative importance of the two addends and was set to 0.7, a value that represents a good trade-off between diffusion rate and computational cost and determined consistent results in previous studies [7,28,56,57,58]. The resulting matrix $X^{ss}$, containing ND scores, was column-wise normalized by the maximum of each column, obtaining the matrix $X^{*}$. Similarly to what was done by Ruffalo et al. [59], a final diffusion score $d_{i}$ was calculated for each gene *i*, multiplying the sum of its three scores $\left(x_{i1}^{*},\;x_{i2}^{*},\;x_{i3}^{*}\right)$ by the sum of the three averages $\left(y_{i1}^{*},\;y_{i2}^{*},\;y_{i3}^{*}\right)$ obtained considering the top 3 direct neighbors of *i* with the highest diffusion scores in each layer [60]. Statistical significance of gene scores was assessed by empirical *p* values, calculated using 1000 permutations of the input matrix $X_{0}$.

### 4.6. Functional Characterization of the INT-MODULE

Topological community identification was performed using methods based on different rationales such as modularity/energy function optimization, edge removal, label propagation, leading eigenvector, and random walks. Modularity was quantified using the Newman definition [61]. Community identification and modularity quantification were performed using functions implemented R package igraph [62].

Pathway analysis was carried out using gene-pathway associations from Biosystems [63] and MSigDB Canonical Pathways [64]. Each pathway was assessed for the over-representation of genes from each cluster using the hypergeometric test (R functions “phyper” and “dhyper”). Nominal *p* values were corrected for multiple testing using the Bonferroni–Hochberg method (R function “p.adjust”), obtaining *q* values.

The enrichment of each cluster in terms of a type *A* of evidence (e.g., genomics) was quantified as the ratio between the fraction of genes supported by *A* in the cluster and the fraction of genes supported by *A* in the INT-MODULE.

## Figures and Tables

**Table 1:** Datasets considered in this study. Selected: The number of genes for which at least a high confidence interaction with any other gene is catalogued in the STRING database (see methods). G: Genomics; E: Epigenomics; T: Transcriptomics. ** major evidence; * minor evidence.

Type of Evidence	Description	Subjects	Number of Genes			
			Initial		Selected	
			**	*	**	*
G	SFARI [8].	-	404	1087	334	799
G	Network diffusion-based prioritization of autism risk genes identifies significantly connected gene modules [7].	-				
G	Meta-analysis of GWAS of over 16,000 individuals with autism spectrum disorder [23].	15,954				
G	Synaptic, transcriptional and chromatin genes disrupted in autism [24].	13,808				
E	Case-control meta-analysis of blood DNA methylation and autism spectrum disorder [10].	1654	416	1444	272	955
T	Gene expression profiling differentiates autism case- controls and phenotypic variants of autism spectrum disorders [25].	116	330	3045	256	2131
T	Blood gene expression signatures distinguish autism spectrum disorders from controls [15].	285				
T	Disrupted functional networks in autism underlie early brain mal-development and provide accurate classification. [26].	147				
T	Gene expression in blood of children with autism spectrum disorder [27].	47				

**Table 2:** The 13 genes that closely interact with the core genes of ASDs. |I|: Number of interactors; |Ic|: Number of interactors that are core genes; p: Hypergeometric probability of observing |Ic| in a hypergeometric experiment; G: Genomics; E: Epigenomics; T: Transcriptomics; ** major; * minor; 0: No evidence; SFARI score: “minimal evidence” (4), “hypothesized but untested” (5); other modules: Reference of gene-networks studies of ASDs in which the gene is mentioned. The total number of genes considered is equal to the interactome size: 12,739 genes.

Symbol	Description	|I|	|Ic|	|core|	*p*	G	E	T	SFARI Score	Other Modules
***HCN4***	hyperpolarization activated cyclic nucleotide gated potassium channel 4	4	2	334	3.97 × 10^−3^	*	*	0	-	-
***DLGAP2***	DLG associated protein 2	21	8	334	3.10 × 10^−8^	*	0	0	4	[18,21,22]
***HCN2***	hyperpolarization activated cyclic nucleotide gated potassium and sodium channel 2	4	1	334	1.01 × 10^−1^	*	0	0	-	-
***UBC***	ubiquitin C	1168	43	334	1.41 × 10^−2^	*	0	0	-	[20]
***NLGN2***	neuroligin 2	28	8	334	4.04 × 10^−7^	*	0	0	4	[18]
***WDR37***	WD repeat domain 37	2	2	334	6.85 × 10^−4^	0	0	*	-	-
***MTMR2***	myotubularin related protein 2	6	1	334	1.47 × 10^−1^	*	0	0	-	-
***EPB41L1***	erythrocyte membrane protein band 4.1 like 1	34	9	334	1.55 × 10^−7^	*	0	0	-	[21]
***GABRA5***	gamma-aminobutyric acid type A receptor alpha5 subunit	17	4	334	8.43 × 10^−4^	*	0	0	5	[21]
***STX1A***	syntaxin 1A	78	10	334	3.47 × 10^−5^	*	0	0	4	[20,21]
***EPB41***	erythrocyte membrane protein band 4.1	16	5	334	4.14 × 10^−5^	*	0	**	-	[20]
***CACNA1F***	calcium voltage-gated channel subunit alpha1 F	37	6	334	3.63 × 10^−4^	*	0	0	4	[21]
***PRKCA***	protein kinase C alpha	197	11	334	1.48 × 10^−2^	*	*	*	4	-

Collectively, we observed that a significant number of “core+13” genes emerged as associated with ASDs at epigenomics level (*p* = 2.63 × 10^−4^; hypergeometic test; Table 3) and at transcriptomics level; *p* = 1.22 × 10^−3^ hypergeometic test, Table 3).

**Table 3:** Overlaps among the lists of genes associated with ASDs. G: Genomic; E: Epigenomics; T: Transcriptomics; ** major; * minor; core+13(E) and core+13(T) indicate genes belonging to the core+13 set and which are supported by E and T, respectively.

A	B	|A|	|B|	|U|	|A∩B|	**〈|A∩B|〉**	P(x≥|A∩B|)
core+13	core+13(E)	347	1227	12739	54	3.27	2.63 × 10^−4^
core+13	core+13(T)	347	2387	12739	88	6.37	1.22 × 10^−3^
G	E	1133	1227	12739	146	109	1.09 × 10^−4^
G	T	1133	2387	12739	235	212	3.95 × 10^−2^
E	T	1227	2387	12739	243	230	1.66 × 10^−1^
G **	E **	334	272	12739	15	7.13	5.47 × 10^−3^
G **	T **	334	256	12739	15	6.71	3.12 × 10^−3^
E **	T **	272	256	12739	5	5.47	6.42 × 10^−1^

**Table 4:** INT-MODULE genes SFARI. G: Genomics; E: Epigenomics; T: Transcriptomics. ** major; * minor. Im: Number of interactors within the INT-MODULE; SFARI score: “minimal evidence” (4), “hypothesized but untested” (5). Other modules: Reference of gene-networks studies that also associated the gene to ASDs.

Symbol	Description	#Im	G	E	T	SFARI Score	Other Modules
***BAIAP2***	BAI1-associated protein 2	4	*	*	0	5	[19,21]
***CACNA1B***	calcium voltage-gated channel subunit alpha1 B	7	0	**	0	4	[19,21]
***CREBBP***	CREB binding protein	43	0	0	**	5	[18,20,21]
***HOXB1***	homeobox B1	12	0	*	*	5	[18]
***INPP1***	inositol polyphosphate-1-phosphatase	1	0	**	0	4	[18,21]
***ITPR1***	inositol 1,4,5-trisphosphate receptor type 1	11	*	*	0	4	[19,21]
***KCNMA1***	potassium large conductance calcium-activated channel, subfamily M, alpha member 1	1	0	**	0	4	[20]
***RASSF5***	Ras association domain family member 5	0	0	**	**	4	-
***RBM8A***	RNA binding motif protein 8A	10	0	**	*	5	-
***SH3KBP1***	SH3-domain kinase binding protein 1	12	*	0	**	5	-

## Supplementary Materials

Supplementary materials can be found at https://www.mdpi.com/1422-0067/20/13/3363/s1.

## References

[1] Schaaf C.P., Zoghbi H.Y. (2011). Solving the autism puzzle a few pieces at a time. Neuron.

[2] Barabási A.L., Gulbahce N., Loscalzo J. (2011). Network medicine: A network-based approach to human disease. Nat. Rev. Genet..

[3] Boyle E.A., Li Y.I., Pritchard J.K. (2017). An Expanded View of Complex Traits: From Polygenic to Omnigenic. Cell.

[4] Devlin B., Scherer S.W. (2012). Genetic architecture in autism spectrum disorder. Curr. Opin. Genet. Dev..

[5] Mitra K., Carvunis A.R., Ramesh S.K., Ideker T. (2013). Integrative approaches for finding modular structure in biological networks. Nat. Rev. Genet..

[6] Cowen L., Ideker T., Raphael J.B., Sharan R. (2017). Network propagation: A universal amplifier of genetic associations. Nat. Rev..

[7] Mosca E., Bersanelli M., Gnocchi M., Moscatelli M., Castellani G., Milanesi L., Mezzelani A. (2017). Network Diffusion-Based Prioritization of Autism Risk Genes Identifies Significantly Connected Gene Modules. Front. Genet..

[8] Abrahams B.S., Arking D.E., Campbell D.B., Mefford H.C., Morrow E.M., Weiss L.A., Menashe I., Wadkins T., Banerjee-Basu S., Packer A. (2013). SFARI Gene 2.0: A community-driven knowledgebase for the autism spectrum disorders (ASDs). Mol. Autism.

[9] Wiśniowiecka-Kowalnik B., Nowakowska B.A. (2019). Genetics and epigenetics of autism spectrum disorder—Current evidence in the field. J. Appl. Genet..

[10] Andrews S.V., Sheppard B., Windham G.C., Schieve L.A., Schendel D.E., Croen L.A., Chopra P., Alisch R.S., Newschaffer C.J., Warren S.T. (2018). Case-control meta-analysis of blood DNA methylation and autism spectrum disorder. Mol. Autism.

[11] Luo R., Sanders S.J., Tian Y., Voineagu I., Huang N., Chu S.H., Klei L., Cai C., Ou J., Lowe J.K. (2012). Genome-wide transcriptome profiling reveals the functional impact of rare de novo and recurrent CNVs in autism spectrum disorders. Am. J. Hum. Genet..

[12] Codina-Solà M., Rodríguez-Santiago B., Homs A., Santoyo J., Rigau M., Aznar-Laín G., Campo M., Gener B., Gabau E., Botella M.P. (2015). Integrated analysis of whole-exome sequencing and transcriptome profiling in males with autism spectrum disorders. Mol. Autism.

[13] Karczewski K.J., Snyder M.P. (2018). Integrative omics for health and disease. Nat. Rev. Genet..

[14] Higdon R., Earl R.K., Stanberry L., Hudac C.M., Montague E., Stewart E., Janko I., Choiniere J., Broomall W., Kolker N. (2015). The promise of multi-omics and clinical data integration to identify and target personalized healthcare approaches in autism spectrum disorders. OMICS.

[15] Kong S.W., Collins C.D., Shimizu-Motohashi Y., Holm I.A., Campbell M.G., Lee I.H., Brewster S.J., Hanson E., Harris H.K., Lowe K.R. (2012). Characteristics and Predictive Value of Blood Transcriptome Signature in Males with Autism Spectrum Disorders. PLoS ONE.

[16] Tylee D.S., Kawaguchi D.M., Glatt S.J. (2013). On the Outside, Looking in: A Review and Evaluation of the Comparability of Blood and Brain “-omes”. Am. J. Med. Genet. Part B.

[17] Andrews S.V., Ellis S.E., Bakulski K.M., Sheppard B., Croen L.A., Hertz-Picciotto I., Newschaffer C.J., Feinberg A.P., Arking D.E., Ladd-Acosta C. (2017). Cross-tissue integration of genetic and epigenetic data offers insight into autism spectrum disorder. Nat. Commun..

[18] Li J., Shi M., Zhihai Ma Z., Zhao S., Euskirchen G., Ziskin J., Urban A., Hallmayer J., Snyder M. (2014). Integrated systems analysis reveals a molecular network underlying autism spectrum disorders. Mol. Syst. Biol..

[19] Gilman S.R., Iossifov I., Levy D., Ronemus M., Wigler M., Vitkup D. (2011). Rare de novo variants associated with autism implicate a large functional network of genes involved in formation and function of synapses. Neuron.

[20] Hormozdiari F., Penn O., Borenstein E., Eichler E.E. (2015). The discovery of integrated gene networks for autism and related disorders. Genome Res..

[21] Parikshak N.N., Luo R., Zhang A., Won H., Lowe J.K., Chandran V., Horvath S., Geschwind D.H. (2013). Integrative functional genomic analyses implicate specific molecular pathways and circuits in autism. Cell.

[22] Pinto D., Delaby E., Merico D., Barbosa M., Merikangas A., Klei L., Thiruvahindrapuram B., Xu X., Ziman R., Wang Z. (2014). Convergence of genes and cellular pathways dysregulated in autism spectrum disorders. Am. J. Hum. Genet..

[23] (2017). The Autism Spectrum Disorders Working Group of the Psychiatric Genomics Consortium. Meta-analysis of GWAS of over 16,000 individuals with autism spectrum disorder highlights a novel locus at 10q24.32 and a significant overlap with schizophrenia. Mol. Autism.

[24] De Rubeis S., He X., Goldberg A.P., Poultney C.S., Samocha K., Cicek A.E., Kou Y., Liu L., Fromer M., Walker S. (2014). Synaptic, transcriptional and chromatin genes disrupted in autism. Nature.

[25] Hu V.W., Sarachana T., Kim K.S., Nguyen A., Kulkarni S., Steinberg M.E., Luu T., Lai Y., Lee N.H. (2009). Gene Expression Profiling Differentiates Autism Case–Controls and Phenotypic Variants of Autism Spectrum Disorders: Evidence for Circadian Rhythm Dysfunction in Severe Autism. Autism Res..

[26] Pramparo T., Lombardo M.V., Campbell K., Barnes C.C., Marinero S., Solso S., Young J., Mayo M., Dale A., Ahrens-Barbeau C. (2015). Cell cycle networks link gene expression dysregulation, mutation, and brain maldevelopment in autistic toddlers. Mol. Syst. Biol..

[27] Gregg J.P., Lit L., Baron C.A., Hertz-Picciotto I., Walker W., Davis R.A., Croen L.A., Ozonoff S., Hansen R., Pessah I.N. (2008). Gene expression changes in children with autism. Genomics.

[28] Bersanelli M., Mosca E., Remondini D., Castellani G., Milanesi L. (2016). Network diffusion-based analysis of high-throughput data for the detection of differentially enriched modules. Sci. Rep..

[29] Yi F., Danko T., Botelho S.C., Patzke C., Pak C., Wernig M., Südhof T.C. (2016). Autism-associated SHANK3 haploinsufficiency causes Ih channelopathy in human neurons. Science.

[30] Zhu M., Idikuda V.K., Wang J., Wei F., Kumar V., Shah N., Waite C.B., Liu Q., Zhou L. (2018). Shank3-deficient thalamocortical neurons show HCN channelopathy and alterations in intrinsic electrical properties. J Physiol..

[31] Nava C., Dalle C., Rastetter A., Striano P., de Kovel C.G., Nabbout R., Cancès C., Ville D., Brilstra E.H., Gobbi G. (2014). De novo mutations in HCN1 cause early infantile epileptic encephalopathy. Nat. Genet..

[32] Feng J., Schroer R., Yan J., Song W., Yang C., Bockholt A., Cook E.H., Skinner C., Schwartz C.E., Sommer S.S. (2006). High frequency of neurexin 1β signal peptide structural variants in patients with autism. Neurosci. Lett..

[33] Gauthier J., Siddiqui T.J., Huashan P., Yokomaku D., Hamdan F.F., Champagne N., Lapointe M., Spiegelman D., Noreau A., Lafrenière R.G. (2011). Truncating mutations in NRXN2 and NRXN1 in autism spectrum disorders and schizophrenia. Hum. Genet..

[34] Parente D.J., Garriga C., Baskin B., Douglas G., Cho M.T., Araujo G.C., Shinawi M. (2017). Neuroligin 2 nonsense variant associated with anxiety, autism, intellectual disability, hyperphagia, and obesity. Am. J. Med. Genet. A.

[35] Jamain S., Quach H., Betancur C., Råstam M., Colineaux C., Gillberg I.C., Soderstrom H., Giros B., Leboyer M., Gillberg C. (2003). Paris Autism Research International Sibpair Study. Mutations of the X-linked genes encoding neuroligins NLGN3 and NLGN4 are associated with autism. Nat. Genet..

[36] Jiang-Xie L.F., Liao H.M., Chen C.H., Chen Y.T., Ho S.Y., Lu D.H., Lee L.J., Liou H.H., Fu W.M., Gau S.S. (2014). Autism-associated gene Dlgap2 mutant mice demonstrate exacerbated aggressive behaviors and orbitofrontal cortex deficits. Mol. Autism.

[37] Marshall C.R., Noor A., Vincent J.B., Lionel A.C., Feuk L., Skaug J., Shago M., Moessner R., Pinto D., Ren Y. (2008). Structural variation of chromosomes in autism spectrum disorder. Am. J. Hum. Genet..

[38] Pinto D., Pagnamenta A.T., Klei L., Anney R., Merico D., Regan R., Conroy J., Magalhaes T.R., Correia C., Abrahams B.S. (2010). Functional impact of global rare copy number variation in autism spectrum disorders. Nature.

[39] Poquet H., Faivre L., El Chehadeh S., Morton J., McMullan D., Hamilton S., Goel H., Isidor B., Le Caignec C., Andrieux J. (2017). Further Evidence for Dlgap2 as Strong Autism Spectrum Disorders/Intellectual Disability Candidate Gene. Autism Open Access.

[40] Nakamura K., Anitha A., Yamada K., Tsujii M., Iwayama Y., Hattori E., Toyota T., Suda S., Takei N., Iwata Y. (2008). Genetic and expression analyses reveal elevated expression of syntaxin 1A (STX1A) in high functioning autism. Int. J. Neuropsychopharmacol..

[41] Nakamura K., Iwata Y., Anitha A., Miyachi T., Toyota T., Yamada S., Tsujii M., Tsuchiya K.J., Iwayama Y., Yamada K. (2011). Replication study of Japanese cohorts supports the role of STX1A in autism susceptibility. Prog. Neuropsychopharmacol. Biol. Psychiatry.

[42] Kofuji T., Hayashi Y., Fujiwara T., Sanada M., Tamaru M., Akagawa K. (2017). A part of patients with autism spectrum disorder has haploidy of HPC-1/syntaxin1A gene that possibly causes behavioral disturbance as in experimentally gene ablated mice. Neurosci. Lett..

[43] Durdiaková J., Warrier V., Banerjee-Basu S., Baron-Cohen S., Chakrabarti B. (2014). STX1A and Asperger syndrome: A replication study. Mol. Autism..

[44] Fatemi S.H., Reutiman T.J., Folsom T.D., Rooney R.J., Patel D.H., Thuras P.D. (2010). mRNA and protein levels for GABAAα4, α5, β1 and GABABR1 receptors are altered in brains from subjects with autism. J. Autism Dev. Disord..

[45] Yang S., Guo X., Dong X., Han Y., Gao L., Su Y., Dai W., Zhang X. (2017). GABA(A) receptor subunit gene polymorphisms predict symptom-based and developmental deficits in Chinese Han children and adolescents with autistic spectrum disorders. Sci. Rep..

[46] Coe B.P., Stessman H.A.F., Sulovari A., Geisheker M.R., Bakken T.E., Lake A.M., Dougherty J.D., Lein E.S., Hormozdiari F., Bernier R.A. (2019). Neurodevelopmental disease genes implicated by de novo mutation and copy number variation morbidity. Nat. Genet..

[47] Sudarov A., Gooden F., Tseng D., Gan W.B., Ross M.E. (2013). Lis1 controls dynamics of neuronal filopodia and spines to impact synaptogenesis and social behaviour. EMBO Mol. Med..

[48] O’Roak B.J., Vives L., Girirajan S., Karakoc E., Krumm N., Coe B.P., Levy R., Ko A., Lee C., Smith J.D. (2012). Sporadic autism exomes reveal a highly interconnected protein network of de novo mutations. Nature.

[49] Iossifov I., Ronemus M., Levy D., Wang Z., Hakker I., Rosenbaum J., Yamrom B., Lee Y.H., Narzisi G., Leotta A. (2012). De novo gene disruptions in children on the autistic spectrum. Neuron.

[50] Griesi-Oliveira K., Acab A., Gupta A.R., Sunaga D.Y., Chailangkarn T., Nicol X., Nunez Y., Walker M.F., Murdoch J.D., Sanders S.J. (2015). Modeling non-syndromic autism and the impact of TRPC6 disruption in human neurons. Mol. Psychiatry.

[51] Beltrão-Braga P.C., Muotri A.R. (2017). Modeling autism spectrum disorders with human neurons. Brain Res..

[52] Szklarczyk D., Franceschini A., Wyder S., Forslund K., Heller D., Huerta-Cepas J., Simonovic M., Roth A., Santos A., Tsafou K.P. (2015). STRING v10: Protein-protein interaction networks, integrated over the tree of life. Nucleic Acids Res..

[53] Brown G.R., Hem V., Katz K.S., Ovetsky M., Wallin C., Ermolaeva O., Tolstoy I., Tatusova T., Pruitt K.D., Maglott D.R. (2015). Gene: A gene-centered information resource at NCBI. Nucleic Acids Res..

[54] Willer C.J., Li Y., Abecasis G.R. (2010). METAL: Fast and efficient meta-analysis of genomewide association scans. Bioinformatics.

[55] Saeliw T., Tangsuwansri C., Thongkorn S., Chonchaiya W., Suphapeetiporn K., Mutirangura A., Tencomnao T., Hu V.W., Sarachana T. (2018). Integrated genome-wide Alu methylation and transcriptome profiling analyses reveal novel epigenetic regulatory networks associated with autism spectrum disorder. Mol. Autism.

[56] Vanunu O., Magger O., Ruppin E., Shlomi T., Sharan R. (2010). Associating Genes and Protein Complexes with Disease via Network Propagation. PLoS Comput. Biol..

[57] Mosca E., Alfieri R., Milanesi L. (2014). Diffusion of Information throughout the Host Interactome Reveals Gene Expression Variations in Network Proximity to Target Proteins of Hepatitis C Virus. PLoS ONE.

[58] Hofree M., Shen J.P., Carter H., Gross A., Ideker T. (2013). Network-based stratification of tumor mutations. Nat. Methods.

[59] Ruffalo M., Koyuturk M., Sharan R. (2015). Network-Based Integration of Disparate Omic Data to Identify “Silent Players” in Cancer. PLoS Comput. Biol..

[60] Di Nanni N., Gnocchi M., Moscatelli M., Milanesi L., Mosca E. Gene relevance based on multiple evidences in complex networks. Bioinformatics.

[61] Newman M.E.J. (2006). Modularity and community structure in networks. Proc. Natl. Acad. Sci. USA.

[62] Csardi G., Nepusz T. (2006). The igraph software package for complex network research. InterJ. Complex Syst..

[63] Geer L.Y., Marchler-Bauer A., Geer R.C., Han L., He J., He S., Liu C., Shi W., Stephen H., Bryant S.H. (2010). The NCBI BioSystems database. Nucleic Acids Res..

[64] Liberzon A., Subramanian A., Pinchback R., Thorvaldsdóttir H., Tamayo P., Mesirov J.P. (2011). Molecular signatures database (MSigDB) 3.0. Bioinformatics.

