# Supplementary material for: Network-Based Integrative Analysis of Genomics, Epigenomics and Transcriptomics in Autism Spectrum Disorders
Source: Int J Mol Sci. 2019 Jul 9;20(13):3363. doi: 10.3390/ijms20133363 (PMC6651137; doi:10.3390/ijms20133363)
Supplement: Supplementary file 1 [file ijms-20-03363-s001.zip › ijms-538370 supplementary/Supplementary_Figures.pdf]

International Journal of *Molecular Sciences*

MDPI

Supplementary Figures

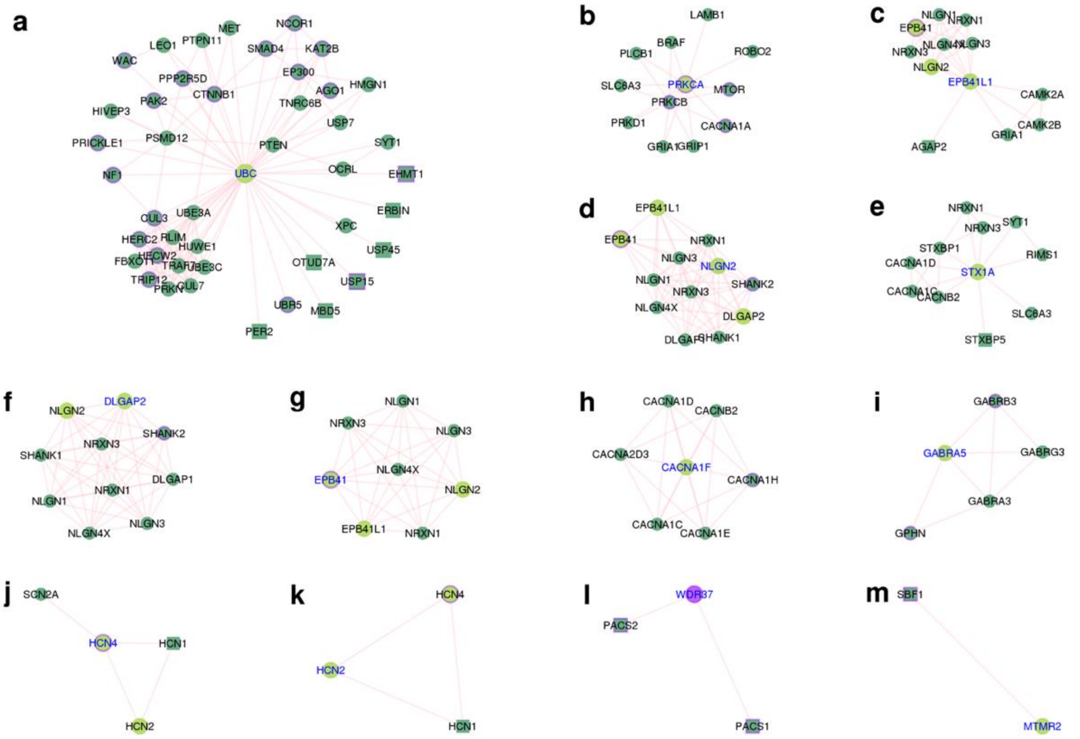

Supplementary Figure S1. Interactors of the 13 genes that lie in network proximity to the CORE genes. The 13 genes are indicated by blue labels. Shape: largest connected component (circle) and other components (square). Fill color: “GENOMICS-MINOR” (light green), SFARI genes (dark green) and no genomics evidence (purple). Border color: epigenetic and/or transcriptomic evidence (purple). Only members of the CORE+13 set are shown, while other direct interactors of the 13 genes are not reported.

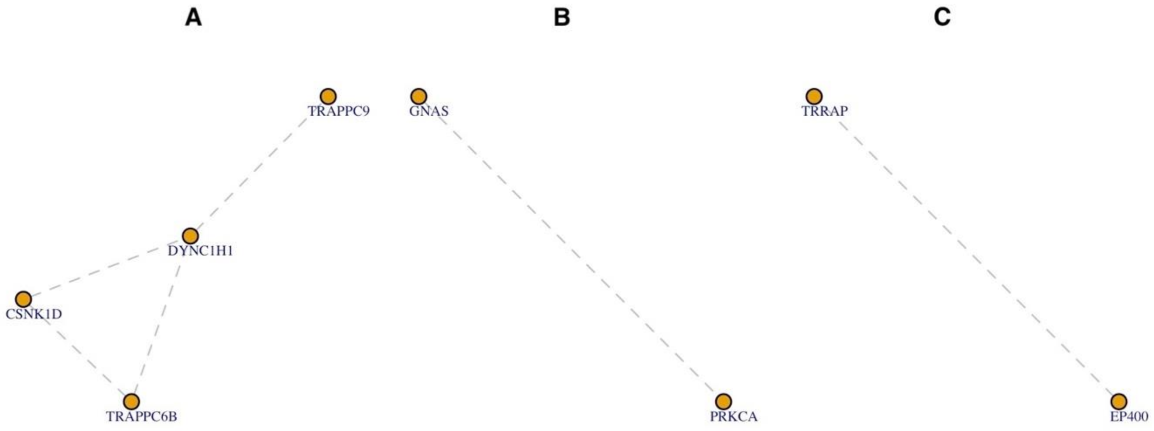

Supplementary Figure S2. Interactions among the SHARED genes. Only 8 of the 34 SHARED genes interact directly with at least another SHARED gene.

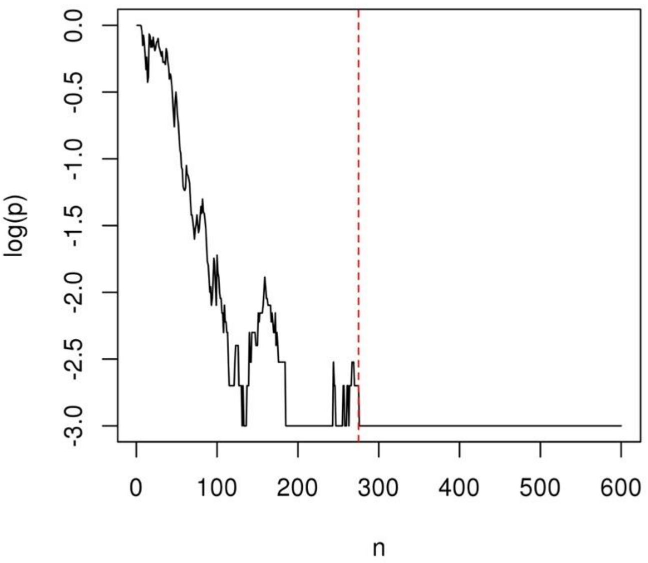

Supplementary Figure S3. Network resampling on the genes top ranked by the multi-omics analysis. Logarithm of *p*-value (y-axis) calculated for each rank of the gene list (x-axis) ordered by decreasing values of the global diffusion score.

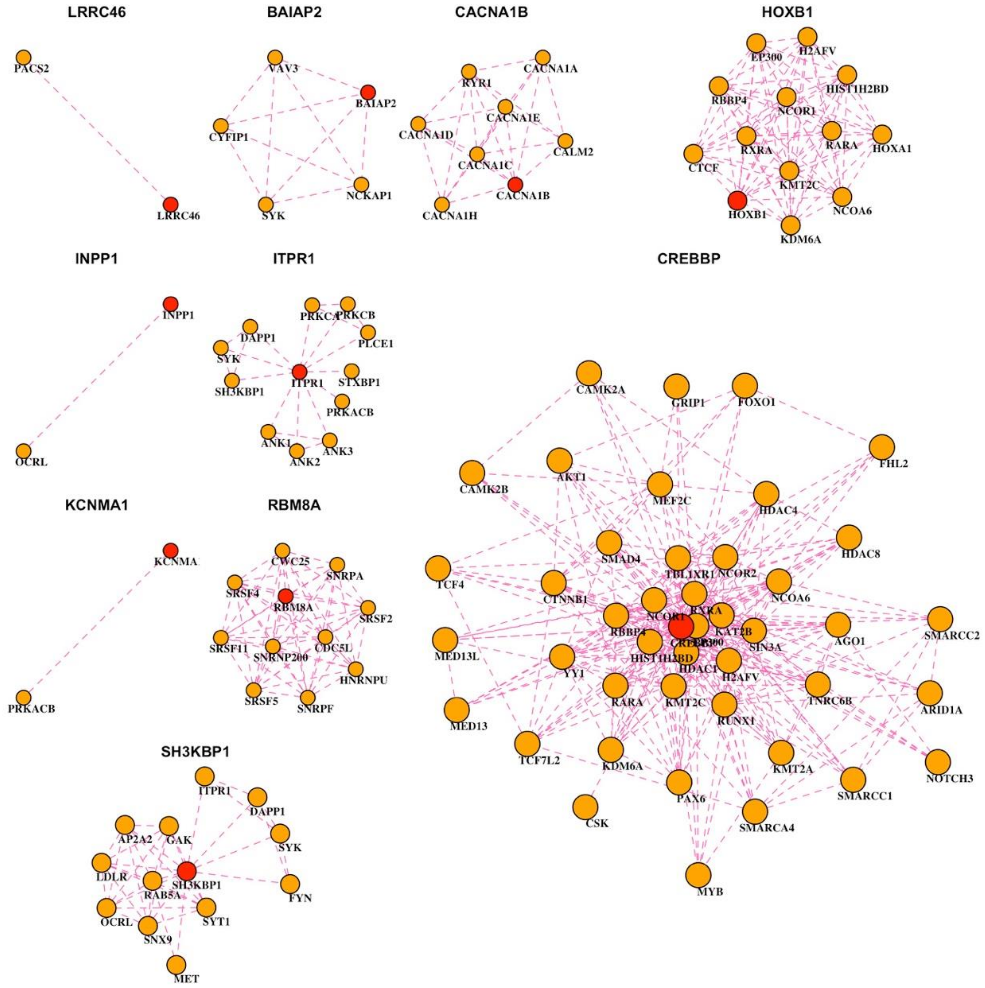

Supplementary Figure S4. Interactors of some genes belonging to the INT-MODULE. Only the interactors that are member of the INT-MODULE are shown, while other interactors are not reported. See main text.

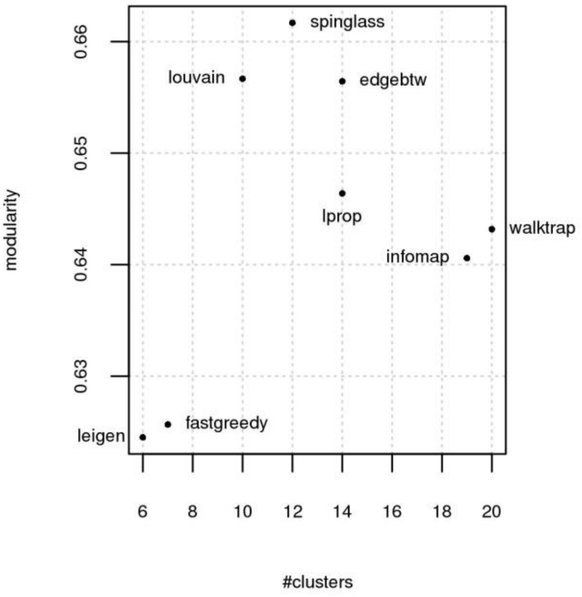

Supplementary Figure S5. Modularity of different partitions of the INT-MODULE. Modularity and number of clusters obtained partitioning the INT-MODULE by means of 8 community detection algorithms.
